# High-temperature NO sensing performance of WO_3_ deposited by spray coating

**DOI:** 10.1039/d2ra02360a

**Published:** 2022-08-09

**Authors:** Roussin Lontio Fomekong, Bilge Saruhan, Marc Debliquy, Driss Lahem

**Affiliations:** Higher Teacher Training College, University of Yaounde I P.O. BOX 47 Yaounde Cameroon lonforou@yahoo.fr; German Aerospace Center (DLR), Institute of Materials Research, Department of High-Temperature and Functional Coatings Cologne 51147 Germany bilge.saruhan@dlr.de; UMONS, Materials Science Department 56, Rue de l’Epargne 7000-Mons Belgium marc.debliquy@umons.ac.be; Materia Nova R&D Center, Materials Science Unit 56, Rue de l’Epargne 7000-Mons Belgium driss.lahem@materianova.be

## Abstract

Nitric oxide (NO) selective sensors capable of sensing in a hot-gas environment are increasingly required for monitoring combustion and processes yielding high temperature gas containing NO. This work reports the fabrication of sensors by a facile deposition of water-based ink blended commercial WO_3_ powders *via* spray coating on sensor platforms fitted with Au-interdigitated electrodes (IDEs) and the characterization of their sensing performances under hot NO-containing air at temperatures exceeding 500 °C. After deposition and heat treatment of the sensing material on the substrate fitted with Au-IDE at 700 °C, the composition and morphology of the active material were analyzed and the presence of a single phase, fine particulates of WO_3_, has been confirmed by XRD and SEM, respectively. The investigation of the sensing properties revealed that, contrary to the previous reports, this WO_3_ sensor can detect NO with a good sensitivity (∼22% for 200 ppm NO) and selectivity at 700 °C under humidity. The effect of relative humidity on sensing performance was also investigated. Also, under humidity values as high as 10% RH and at gas temperatures as high as 700 °C, a reasonably good sensor performance has been observed. It is likely that the improved response towards NO at moderately elevated temperatures resulted from the humidity related water molecules which are adsorbed on the surfaces of WO_3_ particles, providing high affinity hydrogen bonds between NO and OH.

## Abstract 2

Simple WO_3_ has been used for the first time as a semiconductor based gas sensor to detect nitrogen monoxide at high temperature (∼700 °C).

## Graphical abstract

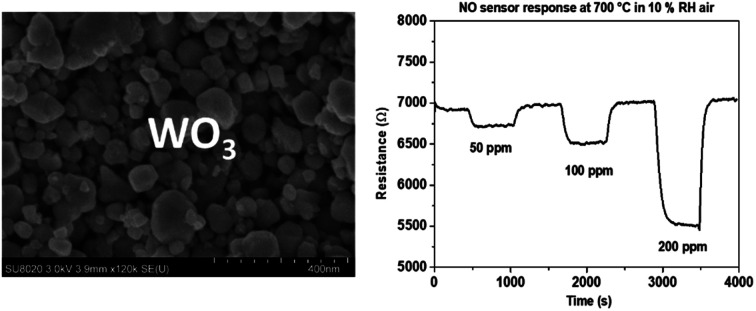

## 1 Introduction

Among noxious pollutants, nitric oxide (NO) is usually formed from fossil fuel combustion which usually involves high temperatures. Due to their superior power and higher efficiency per unit of fuel volume, large diesel vehicles cause a rapid increase of the atmospheric levels of NO. This is because the combustion processes in engines operating at a stoichiometric range oxygen concentration result in the efficient consumption of fuels, which produces a large amount of CO_2_ promoting the formation of NO and NO_2_.^1–3^ The NO emission remains a serious problem worldwide, as it plays an important role in the formation of airpocalypse and acid rain. Therefore, the detection of NO at high temperature becomes very important.^4^ Such high temperature gas sensors capable of operating in harsh environments are needed for utmost safety reasons in general application areas mostly being in the aerospace and space exploration industries.^5^ Moreover, high temperature sensors play a specifically critical role for early fire detection, in the detection of fuel leakage in jet engines, and preventing explosion at combustion engines.^6^

Among the gas detection systems, chemoresistive gas sensors based on semiconductor metal oxides are good candidates due to their several advantages such as simple working principles, greater robustness, relatively low cost, high material sensitivity and real time measurement.^7,8^ However, the optimum sensing temperatures obtained from most of the simple metal oxide (NiO, In_2_O_3_, Cr_2_O_3_, Co_3_O_4_, SnO_2_, *etc.*) are below 400 °C.^9,10^ A literature survey reveals that, Ga_2_O_3_ and TiO_2_ are the mostly applied simple metal oxides for high temperature gas sensing.^11,12^ However, the high resistivity of n-type semiconductor TiO_2_ constitutes a serious limitation for its application as a promising sensing material. Moreover, the stable polymorph of TiO_2_ at high temperature (rutile) is less active as sensor material.^13^ As far as Ga_2_O_3_ is concerned, the oxygen sensors based on β-Ga_2_O_3_ lack in response time and stability at elevated temperatures. Some stable high temperature mixed metal oxides like undoped and doped barium titanate have been used for high temperature NO sensor, but usually, the synthesis of mixed metal oxide system is not easy with the handling of at least two metal sources and can be expensive.^14^

Tungsten trioxide (WO_3_) is a low-cost n-type semiconductor with a band gap of 2.8 eV that exhibits thermal and environmental stability. Apart from the suitable application of nanostructured WO_3_ as photochromic materials, photocatalyst and photoelectrodes, it is one of the best-known gas sensing materials due to its unique chemical and physical characteristic.^15^ Nanostructured tungsten oxide-based gas sensors have been used for detecting a variety of gases, such as NO_2_, NO, CO, H_2_, SO_2_, H_2_, and NH_3_ at moderate temperatures (<500 °C).^16–18^ Although the good sensing properties of WO_3_ were exhibited at moderate temperatures, there is no proper investigation onto its NO-sensing properties at high temperatures (>500 °C).

In the present work, the gas sensing properties of WO_3_ toward NO at moderately elevated temperatures (>500 °C) and under humidity as high as 10% RH are investigated and reported. For that, commercially available WO_3_ nanoparticles were blended in a water-based ink and spray-coated on an Au-IDE fitted sensor platform that are heat-treated and microstructurally characterized for NO-sensing. The results showed that these sensors are capable of selective NO detection in a NO-containing hot synthetic air environment (700 °C). An explanation for the observed enhanced gas sensing performance of WO_3_ based NO sensors is also proposed relying on the presence of humidity, and thus, influence of the water molecule characteristics.

## 2 Materials and methods

### 2.1. Preparation of sensor

In order to perform gas-sensing measurements, the commercial WO_3_ powders (from Sigma Aldrich, Belgium) were deposited as thick films using a spray-coating of the corresponding ink on alumina substrates that were fitted prior with interdigitated Au-electrodes (IDE). The interdigital design is made by 10 gold bars with a gap of 300 μm between each other. Each bar is 2 mm long and 300 μm wide. An eco-friendly water-based ink was used for spray coating of the sensing material. This ink was prepared by vigorously mixing of 10 wt% of metal oxide (*e.g.* WO_3_) powder with distilled water to form a slurry prior to spraying. The spray-coated films were annealed at 700 °C for 1 hour in atmospheric, static air. Before its exposure to gases, the baseline resistance was stabilized by heating the sensor at 700 °C for 1 hour under dry air flow.

### 2.2. Characterization

The identification of the phases was carried out by X-ray diffraction analyses (XRD – Bruker AXS – D8 Advance diffractometer, Kartuizersweg, Belgium) using a Cu K_α1_ source (*λ* = 1.5406 Å).

The morphology of the particles and layers was observed by field emission scanning electron microscopy FE-SEM (Hitachi SU8020 Ultra-High-Resolution Scanning Electron Microscope, Buckinghamshire, United Kingdom) equipped with an EDX detector (Thermo Scientific Ultra dry Noran System 7).

### 2.3. Gas sensing measurement

**Fig. 1:**
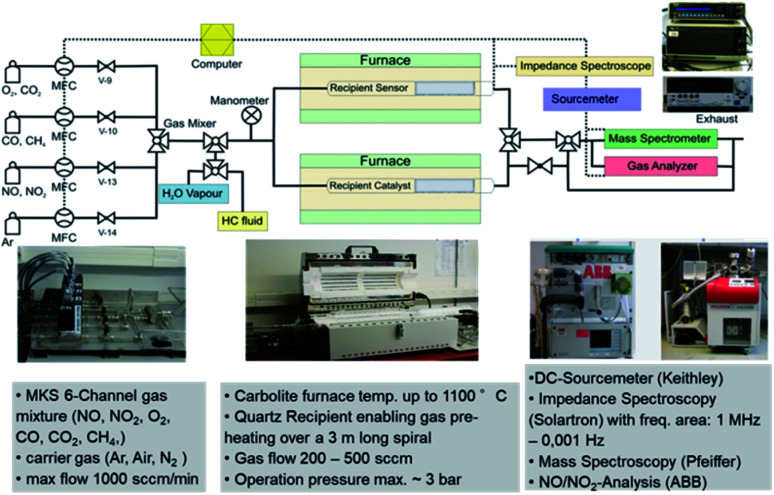
Sensor test equipment at the DLR-Institute of Materials Research in Cologne.

A specially built setup at the DLR-Institute of Materials Research was used to determine the sensors' electrical resistance variation in the presence of target gas(es). The apparatus includes a custom-built quartz glass using as a reactor, affording a thermocouple orientated at the specimen and a cylindrical furnace with cascade control (Fig. 1). A mass flow controller (MFC-647b from MKS Instruments Deutschland GmbH, Munich, Germany) with six-channel was necessary to handle the mixed gas composition. Starting from a 500 ppm gas bottle diluted in N_2_, the NO concentrations were adjusted between 50 and 200 ppm, flowing with a rate of 400 mL min^−1^. The carrier gas used was dry or humid synthetic air composed by 80% N_2_ and 20% O_2_. By bubbling the dry air in water at 50 °C for humidification, a moist air was obtained. The Keithley 2635 A source meter (from Tektronix GmbH Keithley Instruments, Germering, Germany) monitored by a computer Labview program, was employed to perform the DC electrical measurement. Before the fixed amount of the target gas (NO for example) was injected into the chamber for 10 min, the sensor's electrical resistance was first stabilized by putting the sensor in the reference atmospheric air for 10 min. *R*_air_ and *R*_gas_ are the sensor's resistance reached after 10 min in air and in target gas respectively. Since WO_3_ is a n-type semiconductor, the sensor response is given by Δ*R*/*R* × 100. To be more precise, the sensor response can be written as (*R*_air_/*R*_gas_ − 1) × 100 and (*R*_gas_/*R*_air_ − 1) × 100 for reducing and oxidizing gases, respectively. The different measurements were achieved from 600 to 900 °C hot gas.

## 3 Results and discussion

**Fig. 2:**
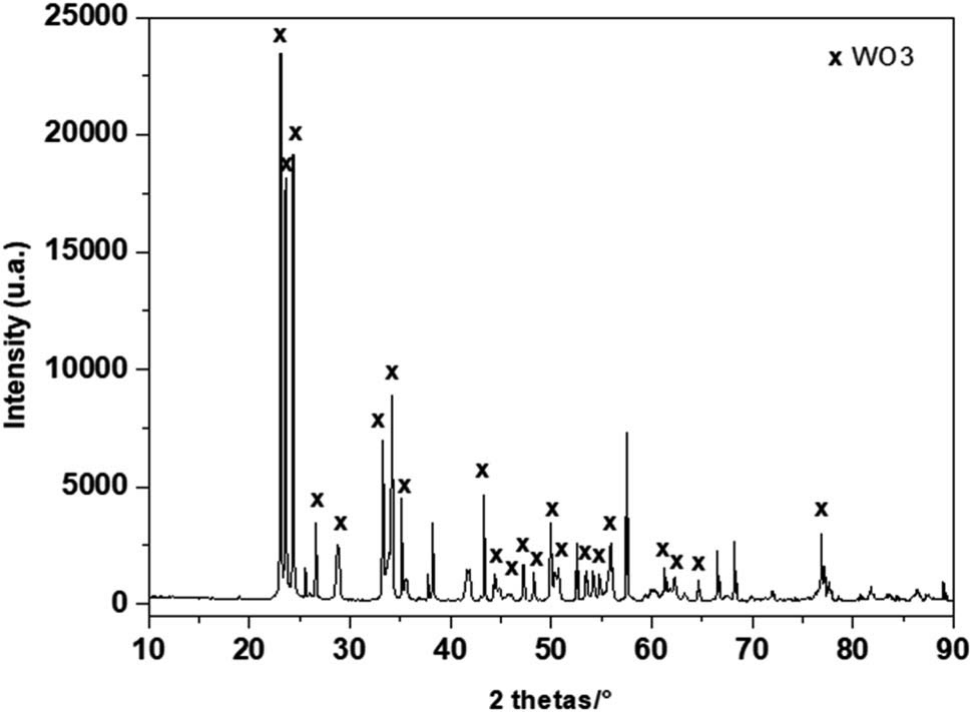
Powder XRD patterns of heat treated WO_3_ layer deposited on alumina substrate fitted with gold interdigital electrodes.

Fig. 2 shows the phase composition investigated by X-ray diffraction (XRD) of the deposited film. The main observed diffraction peaks, (001), (020), (002), (120), (112), (022), (202) and (212), can be indexed as single-phase of monoclinic WO_3_ according to JCPDS card No. 5-0363. The additional diffraction peaks that were detected in the XRD patterns can be assigned to Al_2_O_3_ (the main component of the substrate) and gold from the electrodes.

**Fig. 3:**
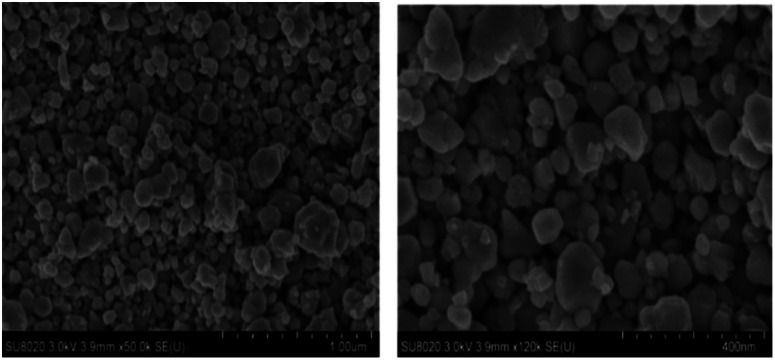
SEM images of heat treated WO_3_ layer coated on alumina substrate fitted with gold interdigital electrodes at different magnifications.

The SEM was used to examine the microstructure of the deposited film. The results show that, the WO_3_ film consists of a quasi-spherical morphology (Fig. 3). Moreover, the film morphology exhibits a quasi-homogenous feature and very little agglomeration with extremely low sintering of particles. This type of morphology with low rigid agglomeration can be a benefit for envisaged sensing applications. The results of semi-quantitative analysis, carried out by EDX confirm the presence of 74.62 at% of O (*cf.* 75 at% expected) and 25.38 at% of W (*cf.* 25 at% expected). No trace of carbon and other elements have been detected, confirming the purity of the sensing material surface.

**Fig. 4:**
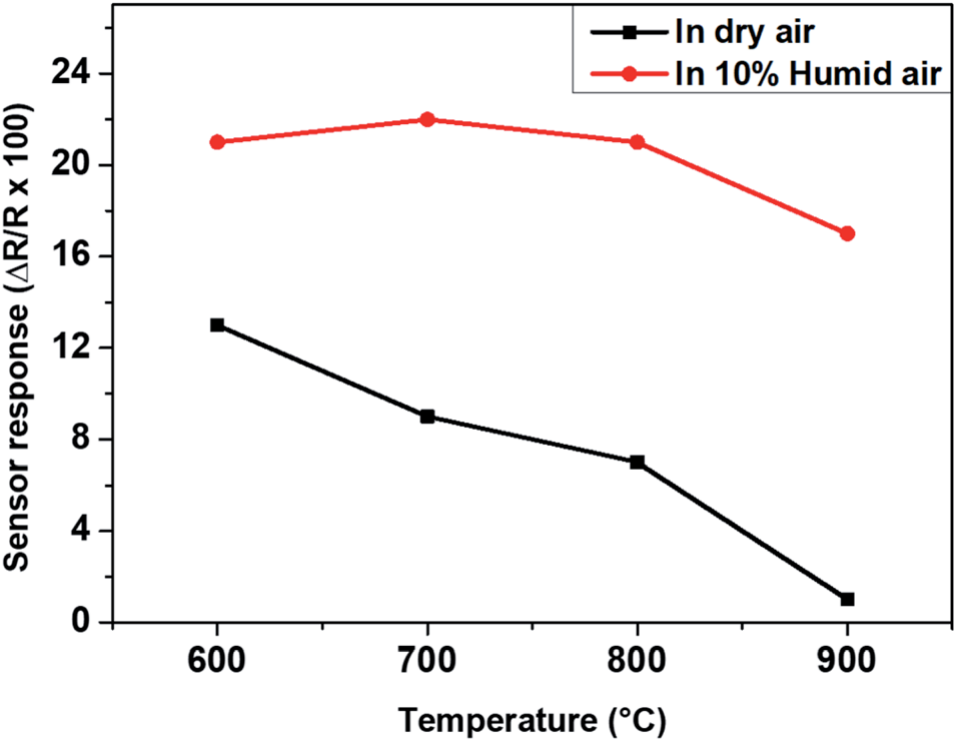
Response of WO_3_ based gas sensor to 200 ppm NO in dry and humid air (set at 50 °C) at different gas temperatures.

The operating temperature is one of the key parameters which influence the sensing response of this type of gas sensor. Humidity is always present in an exhaust gas stream, therefore the responses of the WO_3_ sensor towards 200 ppm of nitrogen monoxide (NO) are tested under dry and humid synthetic air at different temperature (600–900 °C) and given in Fig. 4. The sensor responses are obtained that are 21, 22, 20, 17% in humid air (10% RH), 13, 9, 7 and 1% in dry air at 600, 700, 800 and 900 °C, respectively. In dry synthetic air, the sensor response decreases from 13 to 1% with increasing temperature while, in humid air, the sensor response increases up to 22% at 700 °C before decreasing down to 17% when the temperature further increases. It can be remarked that, the sensor responses are almost twice as higher in the presence of humidity. At high temperature, the hydroxyl group derived from the decomposition of H_2_O vapor, are adsorbed on sensing layer. Given that a good affinity between hydroxyl group and NO exists, due to van der Waals forces, more NO will be consequently adsorbed on the sensing layer and thus, improving of the sensing response in the presence of humidity. A comparable behaviour has been reported by Lontio *et al.* with Rh-doped BaTiO_3_.^14^ However, in the present work, the sensor response obtained at 700 °C with the single phase WO_3_ nanoparticles as sensing layer is higher comparing to what has been reported in the literature (see Table 1), where the highest sensing temperature reached till now with WO_3_ was 350 °C.^16,17^ This is the first time, to the best of our knowledge that WO_3_ clearly detects NO at such a high gas temperature containing humid synthetic air as carrier gas. It is to note that these measurements are carried under hot gas exposure and thus, to differentiate from that of the sensor operating temperature. Relying on this initial result, we have explored intensely further gas sensing properties of this material at 700 °C under NO loaded humid synthetic air.

**Table 1:** Comparison of WO_3_ based gas sensor measurement parameters and results

Synthesis method	Morphology	NO concentration (ppm)	Working temperature (°C)	Response	Ref.
Electrospinning	Nanotubes	5	350	100.3^a^	17
Hydrothermal	Nanowires	500	300	37^a^	20
Bio-template	Microspheres	100	200	46^a^	21
Pyrolysis	Spheres	200	300	23^a^	22
Spray-coating	Nanosphere	200	**700**	22^b^	**This work**

a Gas response *S* = *R*_a_/*R*_g_.

b Gas response *S* = Δ*R*/*R* × 100.

**Fig. 5:**
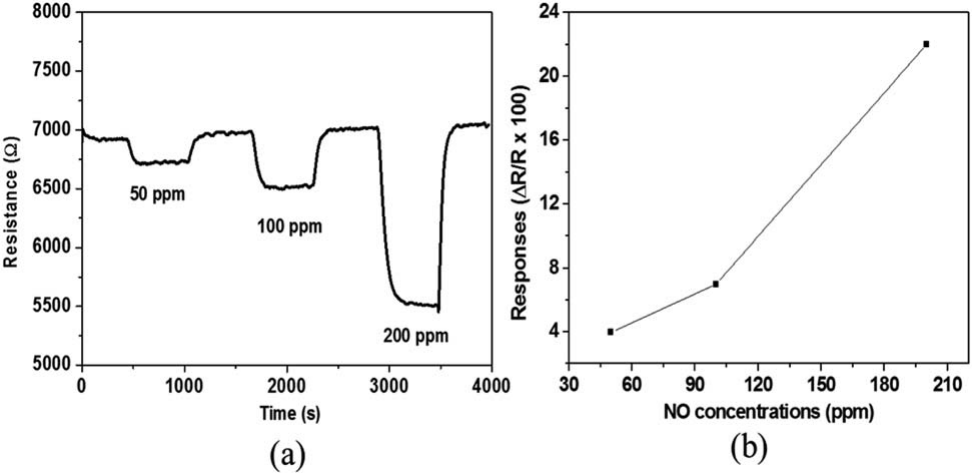
(a) Dynamic curve and (b) response of gas sensors based on WO_3_ in 10% relative humid air (set at 50 °C) to various concentrations of NO at 700 °C.

The measurement of the sensor responses towards different NO concentrations at different gas temperatures in humid air (10% RH) has been performed and the dynamic responses are shown in Fig. 5a. The responses obtained are 4, 7 and 22% for 50, 100 and 200 ppm NO, respectively. As it is observed in Fig. 5b, the sensor response increases with increasing NO concentrations, indicating that the sensor can give a good resolution to the amount of NO in the exhaust gas stream which is very useful for practical application.

**Fig. 6:**
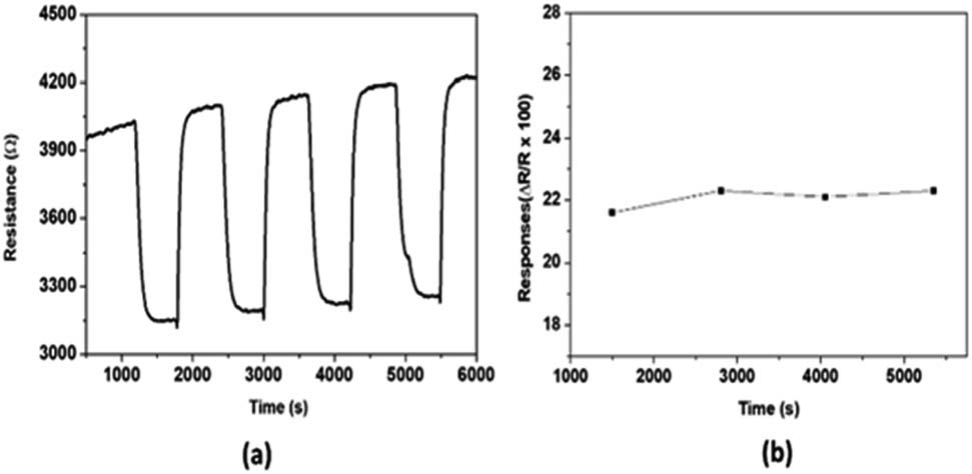
(a) Dynamic curve and (b) response of gas sensors based on WO_3_ to 200 ppm NO at 700 °C.

The repeatability of the sensor response has also been investigated and the results are presented in Fig. 6. After introducing 200 ppm NO successively four times under the same conditions, the sensing responses have been recorded. The results show that, for all the four cycles, after NO injection, the resistance of the sensing layer decreases until the sensor stability is attained, and when the test gas is removed, the resistance returns almost to its initial value. The observed small drift which does not have considerably impact on the repeatability, can be explained by the difficulty of the sensing layer to release the adsorbed NO which is more strongly bonded with the hydroxyl groups present on the WO_3_ surface.

**Fig. 7:**
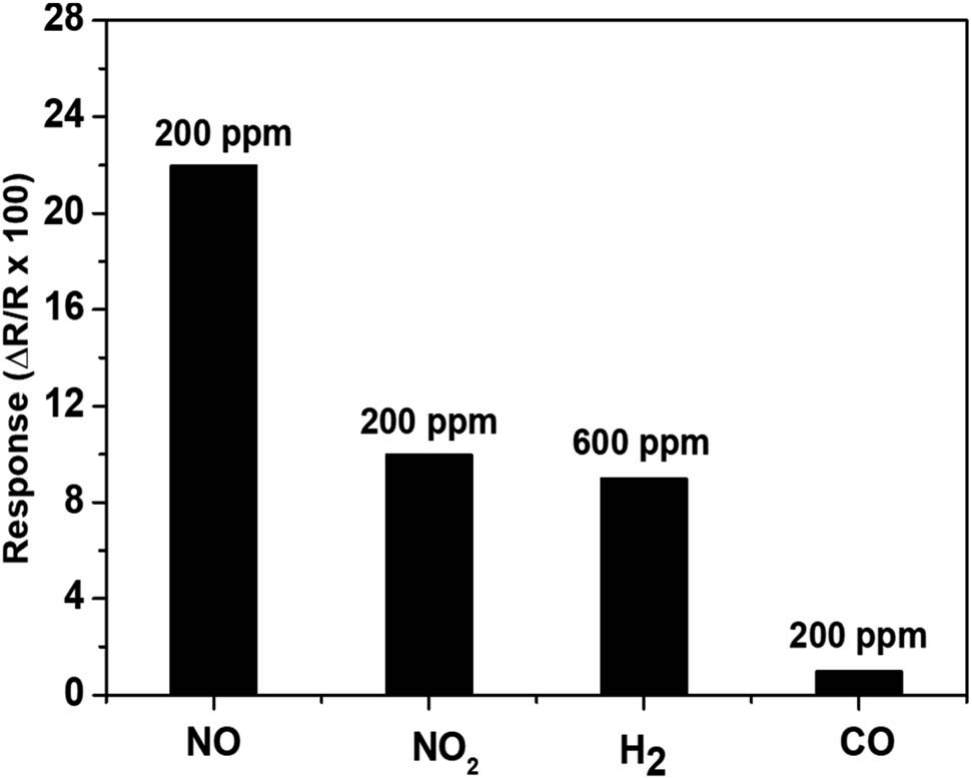
The response of gas sensor based on WO_3_ to various gases including 200 ppm of NO, NO_2_, CO and 600 ppm H_2_ all in 10% relative humid air (set at 50 °C) at 700 °C.

The suitability of a sensor for a practical application should not be limited with its good sensitivity and repeatability, but also be extended to cover its good selectivity towards the target gas. Hence, the selectivity of our sensor towards NO against other potentially interfering gases (CO, NO_2_ and H_2_) has also been investigated. The absolute sensor responses toward 200 ppm of NO_2_, CO, NO and 600 ppm of H_2_ have been measured at 700 °C under 10% of RH and the results are presented in Fig. 7. It should be noted that 600 ppm of H_2_ was the smallest concentration possible in our present set-up. The results indicate that the response to 200 ppm of NO (22%) is more than twice higher than that of NO_2_ (10%), 600 ppm of H_2_ (10%) and 200 ppm of CO (1%). The recorded good NO-selectivity can be attributed to the accomplishment of higher affinity between –OH species and NO rather than other interfering gases. Actually, as the tests are conducted under humidity (*e.g.*, in the presence of water molecules), NO will be promptly adsorbed on the surface considering the fact that the van der Waals forces between O–H and NO are harder than those between O–H and other interfering gases. A similar tendency has been previously reported in the literature with WO_3_ where in the presence of humidity, the sensor response toward CO decreases considerably while the sensor response increases for nitrogen-based compounds (NH_3_ and NO_2_).^19^

**Fig. 8:**
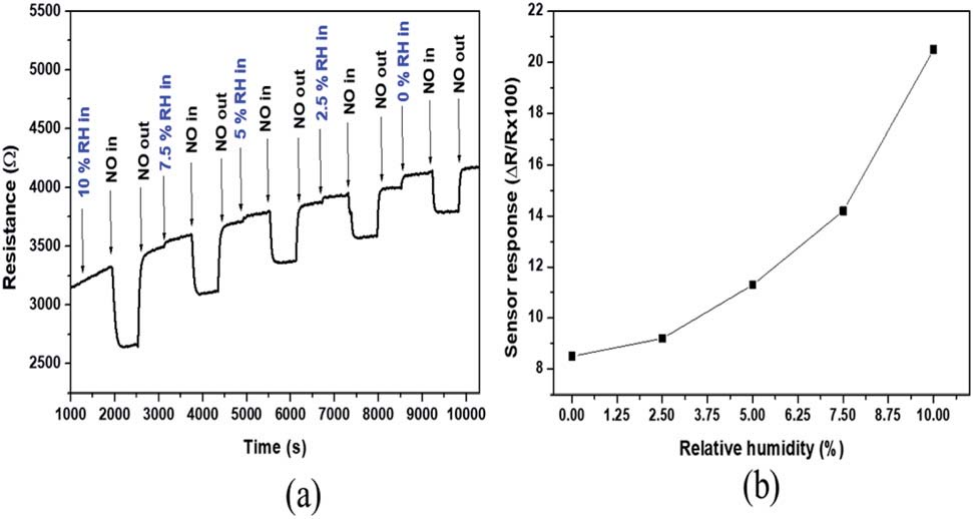
(a) Dynamic curve and (b) response of gas sensors based on WO_3_ to 200 ppm of NO at 700 °C in different relative humidity percentages set at 50 °C (0.0, 2.5, 5.0, 7.5 and 10.0% of RH).

One of the main components of exhaust combustion gases is humidity. Therefore, the humidity effect on NO sensor response has been studied and the results are presented in Fig. 8. The dynamic responses (Fig. 8a) to 200 ppm of NO at various humidity degree (0.0, 2.5, 5.0, 7.5 and 10.0%) have been registered at 700 °C in air. As can be seen in Fig. 8b, the sensor responses are 8, 9, 11, 14 and 21% for 0.0, 2.5, 5.0, 7.5 and 10.0% relative humidity level respectively. The NO sensitivity increases with the increase of humidity level. In fact, following the decomposition of water molecules (related to humidity) at high temperature, the hydroxyl group formed will be adsorbed on the WO_3_ surface. The adsorption of NO will therefore take place *via* the van der Waals forces between O–H and NO. Thus, with the increase of the humidity level, there will be more hydroxyl group adsorbed on the surface, leading to the enhancement of NO-adsorption, and consequently, the increase of sensor response to NO. The drift observed in Fig. 8a can be related to the way that the experiment was carried out. After introducing the target level of humidity, 10 minutes was kept for the stabilisation before 200 ppm of NO was introduced during 10 minutes, and after that, the NO was vented during 10 minutes before the new level of humidity was fixed. In addition to the fact that the baseline resistance increases slightly in the presence of humidity due to the adsorption of OH on material surface, the 10 minutes that was left for the stabilisation after introduction of the target level of humidity was probably not sufficient.

Table 1 compares the temperature at which the optimum sensing was achieved in this work with those reported previously in the literature concerning the detection of NO using WO_3_ based resistive sensor. Despite the difference between the calculation methods of the sensor response, Table 1 illustrates obviously that the present sensing layer performs well at the highest gas temperature of 700 °C and is considerably superior to the most of WO_3_ reported NO-sensors.

### 3.1. Sensing mechanism

**Fig. 9:**
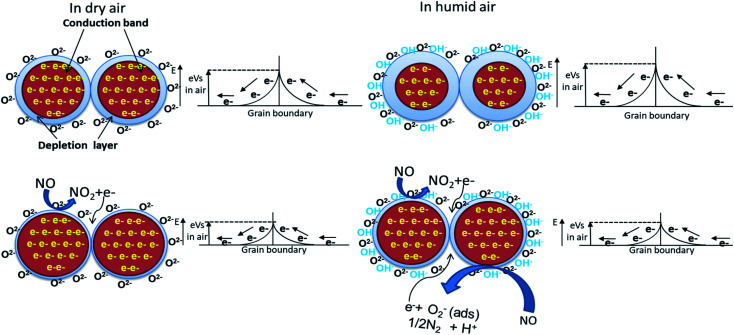
NO-Sensing mechanism with WO_3_ under humidity.

WO_3_ is an n-type semiconductor, and its principle of detection is based on the conductance change in the presence of target gas molecules.^16^ As all the resistive gas sensors, when exposed to synthetic air, the oxygen molecules are directly adsorbed by ionizing (O_2_^−^, O^−^, O^2−^) on the WO_3_ grain surfaces by extracting electrons from the conduction band. The main species at high temperature (above 300 °C) is O^2−^. This generates an electron-depleted space charge layer under the surface, and this provokes the increase of the electrical resistance. After injection of NO, an oxidation reaction with the adsorbed and ionized oxygen will take place [eqn (1)], and the released electrons will return to the conduction band leading to the decrease of thickness of the electron-depleted layer and height of the Schottky barrier and consequently, the resistance of WO_3_ material will decrease (Fig. 9). In the presence of humidity, the adsorption of NO will increase due to the hydroxyl groups' presences (coming from the decomposition of water molecules before reaching the surface) which have a high affinity with NO because of the hydrogen bonds between the two species. Therefore, as more NO is adsorbed on the sensing layer, the enhancement of sensitivity will be observed. A similar effect of water has been reported in the literature.^23^ The increase of sensor signal in the presence of humidity could also be justified by a possibility of a second reaction (between NO and OH–) with the release of an additional electron in the conduction band and this will also increase the sensor response [eqn (2)]. In fact, the reaction is taking place in two steps. The first one is the formation of ionized nitrous acid (HNO_2_^−^) and the second step is its decomposition.

1 NO (gas) + O_ads_^2−^ → NO_2_(gas) + 2e^−^

2 NO + OH^−^ → HNO_2_^−^ → H^+^ + 1/2N_2_ + O_2_^−^(ads) + e^−^

## 4 Conclusions

Commercially available WO_3_ nanoparticles have been deposited by spray coating on substrate fitted with Au-interdigitated electrodes using an eco-friendly water-based ink and heat treated in air at 700 °C for high temperature sensor application. The characterizations of the sensor material phase composition by XRD and EDX-SEM confirm the presence of WO_3_ monoclinic structure and a morphology having the well distributed quasi-spherical particles without agglomeration. For the first time to the best of our knowledge, WO_3_ has been used to efficiently detect NO gas under synthetic humid air at 700 °C. The sensor performances observed were interesting with a good sensor response (22% for 200 ppm of NO under 10% of RH), good selectivity (more than twice as much sensitive to NO than CO, NO_2_ and H_2_) and reasonable reproducibility. The enhancement of sensing properties observed under humidity is probably due to the increase of NO adsorbed on the material surface, relying on the presence of hydroxyl group that are derived from water molecules. This work gives a new orientation on the uses of simple metal oxide such as WO_3_ nanoparticles for high-temperature gas detection.

## Author contributions

Roussin Lontio Fomekong: conceptualization, data curation, methodology and investigation concerning sensor testing, writing original draft. Bilge Saruhan: conceptualization, supervision, project administration, funding acquisition, validation, visualization, writing – review and editing. Marc Debliquy: formal analysis concerning XRD, SEM, writing – review; Driss Lahem: preparation of the sensing layer, writing – review, funding acquisition.

## Conflicts of interest

There are no conflicts to declare.

## Supplementary Material
